# Development and validation of a nomogram for predicting pulmonary complications after video-assisted thoracoscopic surgery in elderly patients with lung cancer

**DOI:** 10.3389/fonc.2023.1265204

**Published:** 2023-10-13

**Authors:** Di Zhao, Anqun Ma, Shuang Li, Jiaming Fan, Tianpei Li, Gongchao Wang

**Affiliations:** ^1^ School of Nursing and Rehabilitation, Shandong University, Jinan, China; ^2^ Department of Thoracic Surgery, Shandong Provincial Hospital Affiliated to Shandong First Medical University, Jinan, China

**Keywords:** after surgery, elderly, lung cancer, nomogram, pulmonary complications

## Abstract

**Background:**

Postoperative pulmonary complications (PPCs) significantly increase the morbidity and mortality in elderly patients with lung cancer. Considering the adverse effects of PPCs, we aimed to derive and validate a nomogram to predict pulmonary complications after video-assisted thoracoscopic surgery in elderly patients with lung cancer and to assist surgeons in optimizing patient-centered treatment plans.

**Methods:**

The study enrolled 854 eligible elderly patients with lung cancer who underwent sub-lobectomy or lobectomy. A clinical prediction model for the probability of PPCs was developed using univariate and multivariate analyses. Furthermore, data from one center were used to derive the model, and data from another were used for external validation. The model’s discriminatory capability, predictive accuracy, and clinical usefulness were assessed using the receiver operating characteristic (ROC) curve, calibration curve, and decision curve analysis, respectively.

**Results:**

Among the eligible elderly patients with lung cancer, 214 (25.06%) developed pulmonary complications after video-assisted thoracoscopic surgery. Age, chronic obstructive pulmonary disease, surgical procedure, operative time, forced expiratory volume in one second, and the carbon monoxide diffusing capacity of the lung were independent predictors of PPCs and were included in the final model. The areas under the ROC curves (AUC) of the training and validation sets were 0.844 and 0.796, respectively. Ten-fold cross-validation was used to evaluate the generalizability of the predictive model, with an average AUC value of 0.839. The calibration curve showed good consistency between the observed and predicted probabilities. The proposed nomogram showed good net benefit with a relatively wide range of threshold probabilities.

**Conclusion:**

A nomogram for elderly patients with lung cancer can be derived using preoperative and intraoperative variables. Our model can also be accessed using the online web server https://pulmonary-disease-predictor.shinyapps.io/dynnomapp/. Combining both may help surgeons as a clinically easy-to-use tool for minimizing the prevalence of pulmonary complications after lung resection in elderly patients.

## 1 Introduction

Based on current sociodemographic trends, lung cancer in older adults is a public health concern, and its magnitude is predicted to peak around 2030 (1). The dramatic increase in lung cancer cases among the geriatric population will put tremendous pressure on surgeons since they may encounter more elderly patients with lung cancer. The National Comprehensive Cancer Network guidelines recommend prioritizing minimally invasive techniques for treatment, such as video-assisted thoracoscopic surgery for stage I and II lung cancers without anatomical and surgical contraindications (2). However, physiological function declines with age, and the capability of older adults (aged 60 years and older) to cope with surgical lung resection decreases. Surgeons must prioritize assessing the hazards of pulmonary and overall outcomes and maintaining the quality of life after surgery.

Postoperative pulmonary complications (PPCs) in elderly patients with lung cancer often occur within 30 days after lung resection (3), with an incidence of 13% to 33% (4–6). Patients with pulmonary complications after surgery have higher morbidity rates and shorter long-term survival than those without pulmonary complications (7–9). In addition, the prevalence of PPCs would prolong the hospitalization time of patients, increase the medical expenses of patients, and exacerbate the financial burden of patients (10–12). Previous studies found that elderly patients undergoing surgical resection have a higher risk of pulmonary complications than younger patients (13, 14). Studies have investigated the risk factors for pulmonary complications after curative resection of early lung cancer in older adults; however, practical evaluation tools for minimizing the occurrence of such PPCs are lacking (15–18). Therefore, it is reasonable to construct and validate an assessment model based on clinical data of elderly patients with lung cancer.

## 2 Patients and methods

### 2.1 Patients selection

**Figure 1:**
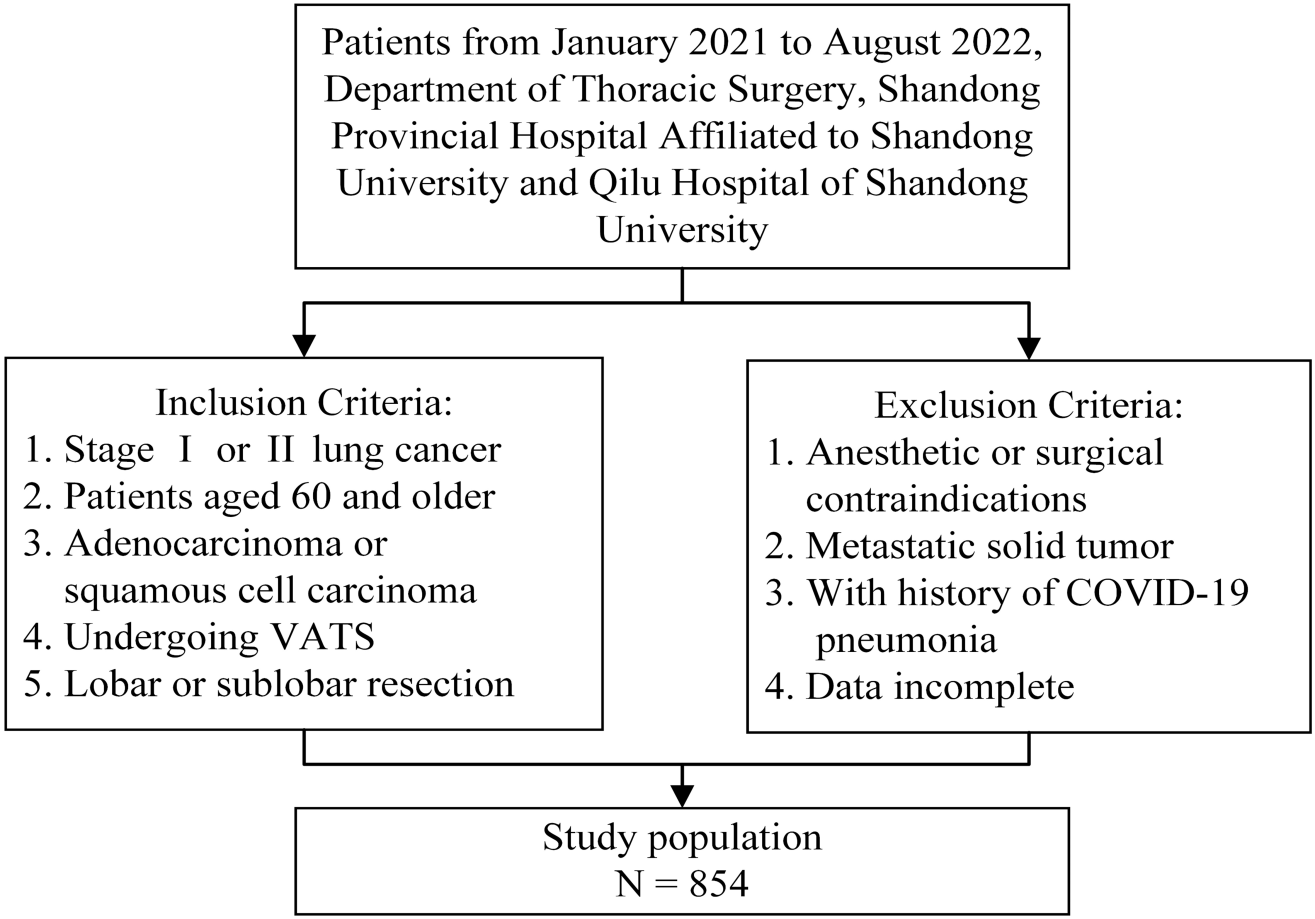
Flow chart of study population screening.

Patients treated between January 2021 and August 2022 at the Department of Thoracic Surgery, Shandong Provincial Hospital Affiliated to Shandong University, and Qilu Hospital of Shandong University were screened if applicable. The primary outcomes were pulmonary complications within 30 days after lung resection. As a composite outcome measure, PPCs included prolonged air leak, pneumonia, pneumothorax, pleural effusion, atelectasis, acute respiratory distress syndrome, bronchopleural fistula, empyema, and respiratory failure (19). Pulmonary complications were assessed using the Clavien-Dindo classification, and the Clavien-Dindo grade ≥ 3 was considered a major complication (20, 21). The participants of this study were patients aged 60 years and older with stage I and II lung cancers. The histological classification criteria were based on the World Health Organization classification of lung tumors, and participants with adenocarcinoma or squamous cell carcinoma of the lungs were included (22). Patients were also selected if they intended to undergo video-assisted thoracoscopic surgery for lobectomy (sleeve lobectomy and bi-lobectomy) or sub-lobar resection (segmentectomy and wedge resection). Patients with contraindications to anesthesia or lung resection and those with severe cardiopulmonary impairment were excluded. Additionally, patients with a history of COVID-19 pneumonia were excluded. Patients with solid metastatic tumors, including in the lymph nodes, were also excluded from the study. Patients and outpatients with incomplete or missing data were not considered. **Figure 1** presents an overview of the inclusion and exclusion criteria. This study conformed to the principles of the Declaration of Helsinki. Because of the anonymity of the data, all cases were exempt from the requirement for individual informed consent. The Institutional Review Board of the School of Nursing and Rehabilitation, Shandong University, approved this study (IRB no. 2022-R-107).

### 2.2 Data collection

Eligible elderly patients with lung cancer were enrolled in this retrospective observational study. Data on the perioperative characteristics were collected. Pre- and intraoperative data include sex, age, smoking history, hypertension, chronic obstructive pulmonary disease (COPD), coronary artery disease, diabetes mellitus, histopathology, tumor location, clinical stage, albumin, hemoglobin, surgical procedures, time of operation, blood loss, forced vital capacity (FVC), forced expiratory volume in one second (FEV1), carbon monoxide diffusing capacity of the lung (DLCO), and so on. All required data were achieved manually from the electronic medical records system. Most variables had no missing data and albumin and hemoglobin levels had a small amount of missing data (<1%), except for FVC (2.2%), FEV1 (10.5%), and DLCO (11.5%). The series mean was used to substitute the missing values for albumin and hemoglobin. Multiple imputations were used to measure FVC, FEV1, and DLCO. Continuous variables were grouped according to actual clinical practice, and categorical variables were encoded prior to logistic regression (**Supplementary Table 1**). Older adults with pulmonary complications were considered “cases” and, if not, “controls”.

The sample size was calculated based on ten events per variable (23), being widely recommended as the minimum standard sample size for logistic regression analysis. In a pre-survey of 50 elderly patients undergoing VATS, PPCs were present in 24% of the patients, comparable to previous studies (13%-33%). This study included 18 pre- and intraoperative variables; therefore, the final sample consisted of 750 elderly patients with lung cancer.

### 2.3 Statistical analysis

Statistical analyses were performed using R for Windows (version 4.2.1; TUNA Team, Tsinghua University, China) and IBM SPSS Statistics software version 27.0. Categorical variables were expressed as frequency (percentage). Continuous variables were expressed as the mean and standard deviation (SD) when distributed normally and as the median and interquartile range (IQR) when non-normally. Pearson’s chi-squared and Fisher’s exact tests (two-sided) were used for statistical tests of categorical variables, Student’s t-test for continuous variables after assessing the normality of data, and the Mann-Whitney U test for non-normal distributions.

Potential correlations between pre- and intraoperative variables were determined using Spearman’s rank correlation. Logistic regression was utilized to analyze the risk factors of pulmonary complications after surgery in 60-year-olds and older with lung cancer. Each variable was initially assessed using univariate analysis. Statistically significant variables (*p*<0.05) were then entered into further multivariate logistic regression analysis. Model construction refers to type 2b transparent reporting of a multivariable prediction model for individual prognosis or diagnosis statement (24). Data from 659 eligible elderly patients with lung cancer from Shandong Provincial Hospital Affiliated to Shandong University were collected for model building, and the data from 195 patients from Qilu Hospital of Shandong University were collected for validation to examine the fitting effect and extrapolation of the predictive model.

We adopted four criteria to evaluate the model performance. First, the receiver operating characteristic (ROC) curve was applied for model discrimination by calculating the area under the ROC curve (AUC) of the test group. Second, a calibration curve was utilized to assess the model calibration, with an outstanding prediction appearing at 45 degrees. Third, the Hosmer-Lemeshow test was employed for goodness-of-fit assessment, indicating an overall fit of the model (*p*>0.05). Fourth, decision curve analysis (DCA) was used to characterize the clinical usefulness and threshold probabilities of the predictive model.

The model was validated utilizing two methods to describe the generalization capability of the predictive model. In the first method, cross-validation was carried out with ten-fold cross-validation. Data from the model building were partitioned into ten equal-sized subsets, and each fold was used as the validation set, and the remaining folds were used as the derived set. Ten-fold cross-validation was performed to avoid overestimating the incremental values because the reported values were the average of running ten-fold cross-validation. In the second method, external data verification was conducted to prevent overfitting and ensure robustness. Data from one center were used for model construction, and data from the other center were used for model validation to test the fit and extrapolation of the predictive model.

## 3 Results

### 3.1 Characteristics of training set and testing set

Among the 854 eligible older adults included in the analysis (**Supplementary Table 2**), data from the Shandong Provincial Hospital Affiliated to Shandong University (n=659) were availed to train the model, and data from the Qilu Hospital of Shandong University (n=195) were availed to test the model. The training and validation sets were further subdivided into controls and cases, and the comparison data for all four sets are shown in **Table 1**. The characteristics of the two groups are presented in **Table 2**. The preoperative and intraoperative characteristics were similar between the training and the test groups, and none of these were significantly different. As shown in **Table 3**, 157 patients had a Clavien-Dindo grade <3, and 142 patients had a Clavien-Dindo grade of 3-5. The most severe pulmonary complications occurred in the participants with multiple complications.

**Table 1:** Comparison of controls with cases for training and testing sets.

Variables	Training set (n=659)		*p*	Testing set (n=195)		*p*
	Controls n=508 (%)	Cases n=151 (%)		Controls n=132 (%)	Cases n=63 (%)	
Sex			<0.001			<0.001
Female	234 (46.1)	95 (62.9)		51 (38.6)	52 (82.5)	
Male	274 (53.9)	56 (37.1)		81 (61.4)	11 (17.5)	
Age, y, median (IQR)	65 (62-68)	68 (63-73)	<0.001	65 (62-68)	68 (62-73)	0.001
Smoking history			0.010			0.026
No	353 (69.5)	88 (58.3)		87 (65.9)	31 (49.2)	
Yes	155 (30.5)	63 (41.7)		45 (34.1)	32 (50.8)	
Hypertension			0.720			0.930
No	341 (67.1)	99 (65.6)		93 (70.5)	44 (69.8)	
Yes	167 (32.9)	52 (34.4)		39 (29.5)	19 (30.2)	
CAD			<0.001			0.890
No	467 (91.9)	123 (81.5)		110 (83.3)	52 (82.5)	
Yes	41 (8.1)	28 (18.5)		22 (16.7)	11 (17.5)	
Diabetes			0.045			0.274
No	449 (88.4)	124 (82.1)		120 (90.9)	54 (85.7)	
Yes	59 (11.6)	27 (17.9)		12 (9.1)	9 (14.3)	
COPD			<0.001			0.048
No	469 (92.3)	91 (60.3)		120 (90.9)	51 (81.0)	
Yes	39 (7.7)	60 (39.7)		12 (9.1)	12 (19.0)	
Tumor site			0.014			0.771
RUL	153 (30.1)	49 (32.5)		48 (36.4)	21 (33.3)	
RML	60 (11.8)	21 (13.9)		8 (6.1)	4 (6.3)	
RLL	79 (15.6)	38 (25.2)		23 (17.4)	16 (25.4)	
LUL	113 (22.2)	22 (14.6)		32 (24.2)	14 (22.2)	
LLL	103 (20.3)	21 (13.9)		21 (15.9)	8 (12.7)	
Histology			<0.001			0.009
ACA	454 (89.4)	117 (77.5)		119 (90.2)	48 (76.2)	
SCA	54 (10.6)	34 (22.5)		13 (9.8)	15 (23.8)	
Clinical stage			0.282			0.726
I a	350 (68.9)	96 (63.6)		95 (72.0)	45 (71.4)	
I b	96 (18.9)	29 (19.2)		24 (18.2)	10 (15.9)	
II a	38 (7.5)	13 (8.6)		7 (5.3)	6 (9.5)	
II b	24 (4.7)	13 (8.6)		6 (4.5)	2 (3.2)	
Surgical procedure			<0.001			<0.001
Sub-lobectomy	269 (53.0)	53 (35.8)		85 (64.4)	12 (19.0)	
Lobectomy	239 (47.0)	97 (64.2)		47 (35.6)	51 (81.0)	
Operative time			<0.001			0.966
<120min	301 (59.3)	41 (27.2)		75 (56.8)	36 (57.1)	
≥120min	207 (40.7)	110 (72.8)		57 (43.2)	27 (42.9)	
Blood loss, mL, median (IQR)	50 (50-80)	80 (50-100)	<0.001	50 (50-80)	80 (50-100)	<0.001
Albumin, g/L, median (IQR)	39 (37-42)	38 (36-41)	0.033	40 (38-42)	38 (36-40)	<0.001
Hemoglobin, g/L, mean (SD)	133 (14.0)	134 (14.5)	0.787	133 (13.6)	131 (15.3)	0.370
FVC, % pred, median (IQR)	103 (94-110)	102 (91-108)	0.110	102 (97-111)	98 (92-108)	0.054
FEV1, % pred, median (IQR)	98 (92-106)	94 (81-104)	<0.001	99 (92-105)	98 (90-103)	0.272
DLCO, % pred, median (IQR)	93 (86-101)	85 (75-91)	<0.001	91(84-101)	88 (80-94)	0.021

CAD, coronary artery disease; COPD, chronic obstructive pulmonary disease; RUL, right upper lobe; RML, right middle lobe; RLL, right lower lobe; LUL, left upper lobe; LLL, left lower lobe; AC, adenocarcinoma; SCA, squamous cell carcinoma; I a, clinical stage Ia; I b, clinical stage Ib; II a, clinical stage II a; II b, clinical stage II b; FVC, forced vital capacity; % pred, percentage of the predicted value; FEV1, forced expiratory volume in one second; DLCO, carbon monoxide diffusing capacity of the lung; SD, standard deviation; IQR, interquartile range.

**Table 2:** Characteristics of the training set and testing set.

Characteristics	Training set n=659	Testing set n=195	*p*
Male, n (%)	330 (50.1)	103 (52.8)	0.501
Age, y, median (IQR)	65 (62-69)	66 (62-68)	0.715
Smoking history yes, n (%)	218 (33.1)	77 (39.5)	0.098
Hypertension yes, n (%)	219 (33.2)	58 (29.7)	0.361
CAD yes, n (%)	69 (10.5)	33 (16.9)	0.015
Diabetes yes, n (%)	86 (13.1)	21 (10.8)	0.398
COPD yes, n (%)	99 (15.0)	24 (12.3)	0.343
Tumor site right upper lobe, n (%)	202 (30.7)	69 (35.4)	0.070
Adenocarcinoma, n (%)	571 (86.6)	167 (85.6)	0.719
Clinical stage I a (%)	446 (67.7)	140 (71.8)	0.695
Lobectomy, n (%)	336 (51.0)	98 (50.3)	0.858
Operative time<120min, n (%)	342 (51.9)	111 (56.9)	0.217
Blood loss, mL, median (IQR)	50 (50-100)	50 (50-100)	0.832
Albumin, g/L, median (IQR)	39 (37-42)	39 (38-41)	0.249
Hemoglobin, g/L, mean (SD)	133 (14.1)	132 (14.2)	0.350
FVC, % pred, median (IQR)	103 (93-110)	101 (94-110)	0.688
FEV1, % pred, median (IQR)	98 (90-105)	99 (92-104)	0.279
DLCO, % pred, median (IQR)	92 (84-99)	89 (81-98)	0.417

CAD, coronary artery disease; COPD, chronic obstructive pulmonary disease; FVC, forced vital capacity; % pred, percentage of the predicted value; FEV1, forced expiratory volume in one second; DLCO, carbon monoxide diffusing capacity of the lung; SD, standard deviation; IQR, interquartile range.

**Table 3:** Classification of pulmonary complications according to Clavien-Dindo grades.

PPCs	Clavien-Dindo grade					Total (%)
	I	II	III	IV	V	
Air leak	0	41	47	0	0	88 (10.3)
Pneumonia	0	33	0	3	0	36 (4.2)
Atelectasis	24	31	41	0	0	96 (11.2)
ARDS/RF	0	0	0	3	0	3 (0.4)
Pneumothorax	13	0	23	0	0	36 (4.2)
Pleural effusion	0	15	18	4	0	37 (4.3)
BPF/empyema	0	0	0	2	1	3 (0.4)
Total (%)	37 (4.3)	120 (14.1)	129 (15.1)	12 (1.4)	1 (0.1)	299 (35.0)

PPCs, postoperative pulmonary complications; ARDS, acute respiratory distress syndrome; RF, respiratory failure; BPF, bronchopleural fistula.

**Figure 2:**
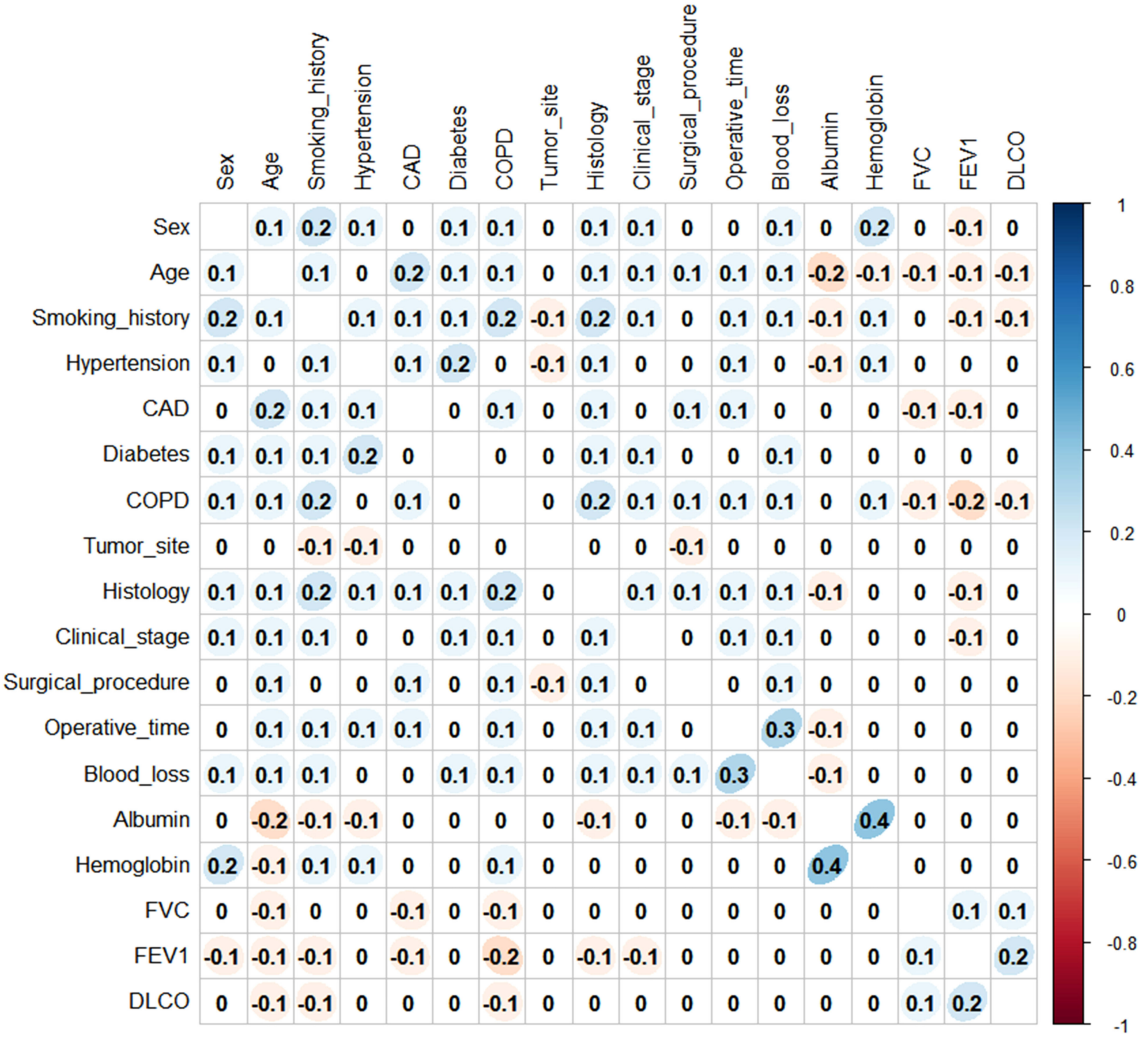
Spearman’s rank correlation of all variables.

Before establishing the nomogram, the preoperative and intraoperative variables were tested for correlation using Spearman’s coefficient. All variables had a low correlation (Spearman’s rho coefficient<0.5; *p*<0.001) and were retained in the subsequent analysis (**Figure 2**).

### 3.2 Building nomogram in training set

As shown in **Table 4**, univariate logistic regression was performed to compare 508 controls and 151 cases in the training set. Age, sex, smoking status, coronary artery disease, diabetes mellitus, COPD, histology, surgical procedure, operative time, blood loss, albumin, FVC, FEV1, and DLCO correlated significantly with PPCs (*p*<0.05). Statistically significant variables were included for further analyses.

**Table 4:** Univariable and multivariable logistic regression analysis.

Variable	Univariate analysis		Multivariate analysis	
	OR (95%CI)	*p*	OR (95%CI)	*p*
Sex	1.986 (1.367-2.886)	<0.001	1.231 (0.704-2.151)	0.466
Age≥70 y	4.783 (3.198-7.153)	<0.001	3.645 (2.043-6.505)	<0.001
Smoking history	1.630 (1.121-2.371)	0.011	0.736 (0.411-1.319)	0.303
Hypertension	1.073 (0.731-1.574)	0.720	−	−
CAD	2.593 (1.542-4.361)	<0.001	0.960 (0.449-2.052)	0.916
Diabetes	1.657 (1.008-2.724)	0.046	0.992 (0.479-2.054)	0.983
COPD	7.929 (4.998-12.578)	<0.001	6.433 (3.389-12.211)	<0.001
Tumor site	0.887 (0.785-1.002)	0.053	−	−
Histology	2.443 (1.520-3.928)	<0.001	0.981 (0.489-1.969)	0.957
Clinical stage	1.198 (0.981-1.462)	0.076	−	−
Surgical procedure	2.022 (1.389-2.944)	<0.001	2.750 (1.632-4.634)	<0.001
Operative time	3.901 (2.615-5.820)	<0.001	3.704 (2.136-6.423)	<0.001
Blood loss, 10 mL	1.132 (1.080-1.186)	<0.001	1.051 (0.998-1.107)	0.059
Albumin<40 g/L	1.525 (1.037-2.243)	0.032	1.205 (0.708-2.053)	0.492
Hemoglobin<120 g/L	1.011 (0.629-1.625)	0.963	−	−
FVC<80%	2.435 (1.173-5.056)	0.017	1.849 (0.611-5.593)	2.276
FEV1<80%	6.836 (3.771-12.392)	<0.001	2.836 (1.299-6.190)	0.009
DLCO<80%	3.585 (2.288-5.615)	<0.001	3.753 (2.073-6.794)	<0.001

CAD, coronary artery disease; COPD, chronic obstructive pulmonary disease; FVC, forced vital capacity; % pred, percentage of the predicted value; FEV1, forced expiratory volume in one second; DLCO, carbon monoxide diffusing capacity of the lung; OR, odds ratio; CI, confidence interval; -, statistically unsignificant values in univariate analysis.

Multivariate regression analysis showed that age, COPD, surgical procedure, operative time, FEV1, and DLCO were independent predictors of PPCs. Patients over or equal to 70 years of age had a 264.5% increased risk of PPCs compared to patients between 60 and 69 years. Patients undergoing lobectomy had a 175.0% greater risk of developing PPCs compared with those undergoing sub-lobectomy. The odds ratio (OR) of operative time equal to or greater than 120 minutes to those less than 120 minutes is 3.704, and the odds ratio of COPD diagnosis to those without is 6.443. Besides, FEV1 (<80% *vs*.≥80%) was the risk factor of PPCs (OR=2.836), and DLCO (<80% *vs*.≥80%) was also the risk factor of PPCs (OR=3.753).

**Figure 3:**
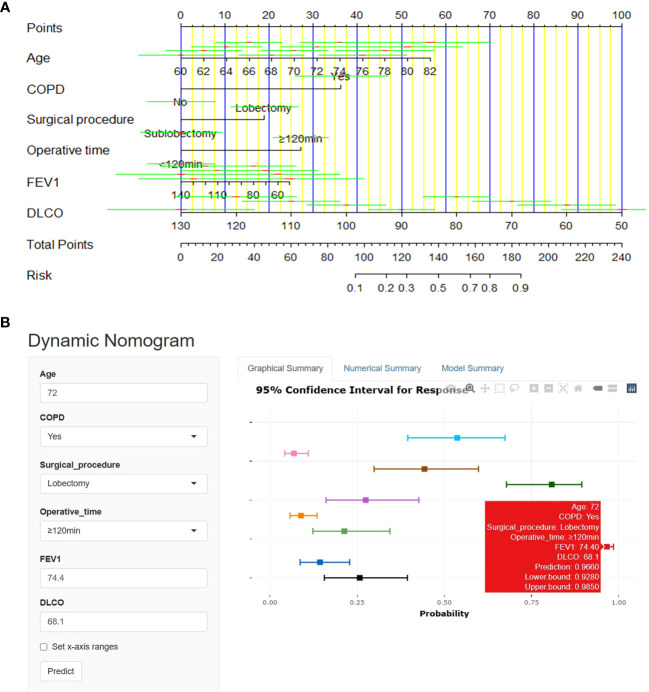
Nomogram for prediction of PPCs in elderly lung cancer patients. **(A)** Use the linear nomogram to predict PPCs. **(B)** Online web-server interface based on the proposed nomogram.

A nomogram was derived to predict the probability of PPCs in elderly patients with lung cancer (**Figure 3A**). After performing univariate and multivariate logistic regression analyses to eliminate potential redundancy, the predicted probability was calculated by summing the scores for each item using a nomogram.

Our dynamic nomogram is available online at https://pulmonary-disease-predictor.shinyapps.io/dynnomapp/as a clinically easy-to-use tool to assist surgeons (**Figure 3B**). Whether or not pulmonary complications have occurred can be expediently judged with little effort by reading information related to the clinical characteristics, and the network server generates the results.

### 3.3 Validation of nomogram

**Figure 4:**
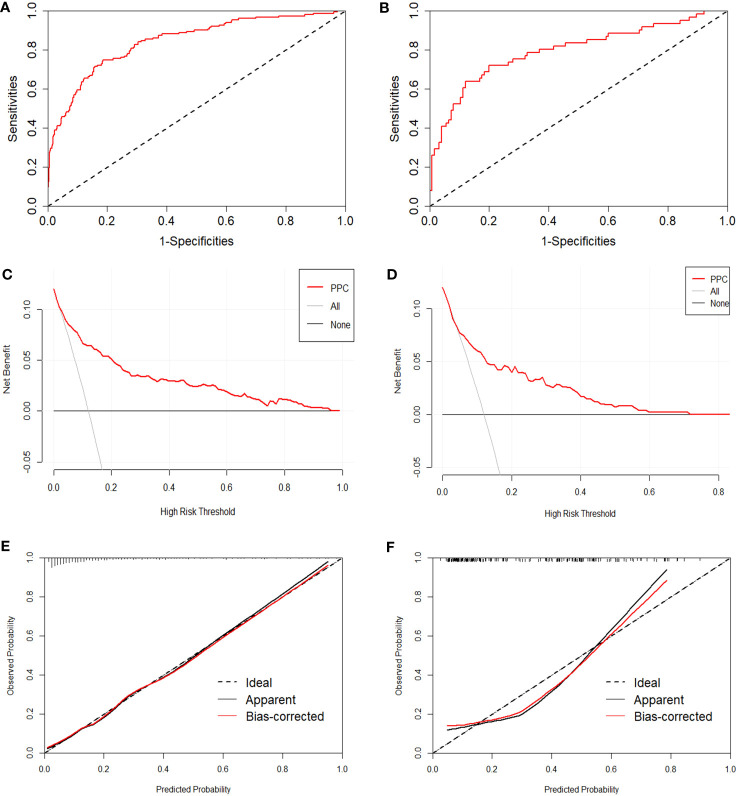
Evaluation of the proposed nomogram in the training and test sets. **(A, B)** ROC curves with false positive rate shown on the x-axis; true positive rate shown on the y-axis. **(C, D)** Calibration curves, with a predicted probability of postoperative pulmonary complications shown on the x-axis and actual probability shown on the y-axis. **(E, F)** Decision curve analysis, presenting threshold probability on the x-axis and net benefit on the y-axis.

Four methods are available for evaluating prediction performance. The first method used all data to perform ten-fold cross-validation to characterize the generalizability of the nomogram. An AUC value of 0.839 (sensitivity=0.949, specificity=0.450) was the average value assessed by ten-fold cross-validation. The second method employs the results of the multivariate regression analysis to calculate the area under the ROC curve. The AUC and 95% confidence interval (CI) of the training set was 0.844 (95%CI: 0.806-0.882, *p*<0.001, **Figure 4A**), and the AUC value of the test set was 0.796 (95% CI: 0.722-0.870, *p*<0.001, **Figure 4B**), both of which fully embody the high discrimination of the nomogram. The third method combines the calibration curve and the Hosmer-Lemeshow test to evaluate the accuracy of the nomogram. The calibration curve visualizes the Hosmer-Lemeshow goodness-of-fit test (*p*>0.05). Calibration curves were constructed to compare the predicted and observed probabilities (**Figures 4C, D**). The Hosmer-Lemeshow goodness-of-fit for the training set was 0.099, and the result for the test set was 0.112. The fourth method is DCA, which showed that the nomogram had superior net benefits, with threshold probabilities ranging from 4%-97% and 6%-71% in **Figures 4E, F**, respectively.

## 4 Discussion

To our knowledge, surgery for lung cancer may damage the lung tissue and increase the risk of pulmonary complications and surgical outcomes (25, 26). Cumulative evidence has demonstrated that pulmonary complications after video-assisted thoracoscopic surgery are the most common postoperative complication, with a worse prognosis and increased readmission, morbidity, and mortality (7, 14, 27, 28).

In this retrospective study, the prevalence of PPCs in elderly patients with lung cancer was 25.06%. After univariate analysis and multivariate logistic regression to eliminate potential redundancies, age, COPD, surgical procedure, operative time, FEV1, and DLCO were included in the final nomogram. We found that older adults who suffered from a curative-intent lung cancer surgery had an almost 264.5% higher incidence rate for patients aged ≥70 years compared to those aged 60-69. This result has far-reaching implications for the current issue of population aging. Lobar and sub-lobar resections in older adults have proven to be vital predictors of pulmonary and overall outcomes, consistent with previous studies (29–31). This study may have driven the perception (32, 33) that COPD diagnosis is associated with PPCs. PPCs are closely relevant to the operative time, and longer operation duration (≥120 min) was associated with severe postoperative complications such as pulmonary complications (34). The percentages of predicted value for FEV1 and DLCO correlated closely with PPCs, in accordance with previous studies (35, 36).

A few of these are designed to treat pulmonary complications in older adults, especially in patients undergoing VATS. Only three studies have noted the risk factors for postoperative complications and the occurrence of pulmonary complications; however, the established risk assessment system for calculating the risk value is not simple or intuitive (15, 17, 35). Current research on pulmonary complications after lung cancer surgery in older adults lacks specific, particularly those focusing on evaluation instruments. A nomogram model of postoperative pulmonary complications in elderly patients undergoing video-assisted thoracoscopic surgery is yet to be developed.

This study used Spearman’s coefficient to analyze the potential correlations between pre- and intraoperative variables and established a nomogram based on univariate and multivariate regression analyses. Furthermore, the proposed nomogram was implemented on an online web server as a clinically easy-to-use tool for guiding surgeons in optimizing patient-centered treatment plans. As expected, older adults (≥70 years old) undergoing lobectomy and diagnosed with COPD, the percentage of the predicted value for FEV1 and DLCO <80%, with operative time ≥120 minutes, had the highest risk (**Figures 3A, B**). Individualized nomogram assessment based on clinical variables could help select video-assisted thoracoscopic surgery patients and differentiate patients at a high risk of pulmonary complications.

The area under the ROC curve for the ten-fold cross-validation was 0.839, indicating an acceptable generalization ability of the prediction model. The ROC curve of this model indicated good discrimination in the training and validation sets (0.884 and 0.796, respectively), which were greater than those of previously published models (Foster et al. (37) found a lower value of 0.65). The Hosmer-Lemeshow goodness-of-fit (*p*>0.05) in the model building and testing sets also showed good consistency in predicting pulmonary complications after surgery, combining the predicted and observed probability shown by the calibration curve. Furthermore, decision curve analysis showed a relatively high level of clinical benefit.

This study has certain limitations. The retrospective and observational nature of the study may have caused bias. However, the data on patient perioperative variables observed are objective, and reliable to avoid information bias from the source of demographics and patients. In addition, we presented only 18 pre- and intraoperative variables in elderly patients with lung cancer. In future studies, we expect to incorporate additional perioperative clinical, surgical, and radiographic data into the new modified model. Furthermore, the data gathered in this study came from two centers, and more data from other medical centers and different population studies are our future research directions. Although the predictive nomogram and test obtained high levels of statistical significance, the importance of the actual clinical benefits will emerge only in practice.

## 5 Conclusions

The incidence of pulmonary complications after video-assisted thoracoscopic surgery in elderly patients with lung cancer was 25.06%. We built and validated a predictive nomogram for PPCs and implemented it on an online web server. A combination of both may assist surgeons in minimizing the risk of pulmonary complications and guide them in optimizing surgical outcomes after lung resection in elderly patients.

## Data availability statement

The original contributions presented in the study are included in the article/**Supplementary Material**. Further inquiries can be directed to the corresponding author.

## Ethics statement

The studies involving humans were approved by the Institutional Review Board of the School of Nursing and Rehabilitation of Shandong University. The studies were conducted in accordance with the local legislation and institutional requirements. The ethics committee/institutional review board waived the requirement of written informed consent for participation from the participants or the participants’ legal guardians/next of kin because the anonymity of the data, informed consent was waived.

## Author contributions

DZ: Conceptualization, Investigation, Project administration, Resources, Supervision, Writing – review & editing, Data curation, Formal Analysis, Methodology, Software, Validation, Visualization, Writing – original draft. AM: Data curation, Investigation, Resources, Writing – original draft. SL: Conceptualization, Formal Analysis, Methodology, Software, Validation, Visualization, Writing – review & editing. JF: Writing – review & editing, Data curation, Investigation. TL: Data curation, Investigation, Writing – review & editing. GW: Investigation, Writing – review & editing, Conceptualization, Funding acquisition, Project administration, Resources, Supervision.
