# Supplementary material for: Development and validation of a nomogram for predicting pulmonary complications after video-assisted thoracoscopic surgery in elderly patients with lung cancer
Source: Front Oncol. 2023 Oct 13;13:1265204. doi: 10.3389/fonc.2023.1265204 (PMC10613030; doi:10.3389/fonc.2023.1265204)
Supplement: Supplementary file 1 [file Table_1.docx]

**Supplementary Table 1** Assignment of variables.

| **Variables** | **Assignment** |
| --- | --- |
| PPC | Controls = 0, Cases = 1 |
| Sex | Female = 0, Male = 1 |
| Age, y | 60-69 = 0, ≥70 =1 |
| Smoking history | Never = 0, Ever = 1 |
| Hypertension | No = 0, Yes = 1 |
| Coronary artery disease | No = 0, Yes = 1 |
| Diabetes | No = 0, Yes = 1 |
| COPD | No = 0, Yes = 1 |
| Tumor site | RUL = 0, RML = 1, RLL = 2,  LUL = 3, LLL = 4 |
| Histology | Adenocarcinoma = 0, Squamous cell carcinoma = 1 |
| Clinical stage | Ⅰ a = 0, Ⅰ b = 1, Ⅱ a = 2, Ⅱ b = 3 |
| Surgical procedure | Sublobectomy = 0, Lobectomy = 1 |
| Operative time, min | <120 = 0, ≥120 = 1 |
| Albumin, g/L | ≥40 = 0, <40 = 1 |
| Hemoglobin, g/L | ≥120 = 0, <120 = 1 |
| FVC, %pred | ≥80 = 0, <80 = 1 |
| FEV1, %pred | ≥80 = 0, <80 = 1 |
| DLCO, %pred | ≥80 = 0, <80 = 1 |

PPC, postoperative pulmonary complication; COPD, chronic obstructive pulmonary disease; RUL, right upper lobe; RML, right middle lobe; RLL, right lower lobe; LUL, left upper lobe; LLL, left lower lobe; FVC, forced vital capacity; % pred, percentage of the predicted value; FEV1, forced expiratory volume in one second; DLCO, carbon monoxide diffusing capacity of the lung.
