# Supplementary material for: Development and validation of a nomogram for predicting pulmonary complications after video-assisted thoracoscopic surgery in elderly patients with lung cancer
Source: Front Oncol. 2023 Oct 13;13:1265204. doi: 10.3389/fonc.2023.1265204 (PMC10613030; doi:10.3389/fonc.2023.1265204)
Supplement: Supplementary file 2 [file Table_2.docx]

**Supplementary Table 2** Characteristics of all data summary.

| **Characteristics** | **All** |
| --- | --- |
| Total | 854 |
| Female: Male | 421: 433 |
| Age, y, median (IQR) | 65 (62-69) |
| Smoking history yes (%) | 295 (34.5) |
| Hypertension yes (%) | 277 (32.4) |
| Coronary artery disease yes (%) | 102 (11.9) |
| Diabetes yes (%) | 107 (12.5) |
| COPD yes (%) | 123 (14.4) |
| Tumor site right upper lobe (%) | 271 (31.7) |
| Adenocarcinoma: Squamous cell carcinoma | 738: 116 |
| Clinical stage Ⅰ a (%) | 586 (68.6) |
| Sublobectomy: Lobectomy | 420: 434 |
| Operative time < 120min (%) | 453 (53.0) |
| Blood loss, mL, median (IQR) | 50 (50-100) |
| Albumin, g/L, median (IQR) | 39 (37-42) |
| Hemoglobin, g/L, mean (SD) | 133 (14.1) |
| FVC, % pred, median (IQR) | 102 (94-110) |
| FEV1, % pred, median (IQR) | 98 (91-105) |
| DLCO, % pred, median (IQR) | 91 (93-99) |

COPD, chronic obstructive pulmonary disease; FVC, forced vital capacity; % pred, percentage of the predicted value; FEV1, forced expiratory volume in one second; DLCO, carbon monoxide diffusing capacity of the lung; SD, standard deviation; IQR, interquartile range.
